# Deorphanizing NUDIX hydrolases from *Trypanosoma*: tantalizing links with metabolic regulation and stress tolerance

**DOI:** 10.1042/BSR20191369

**Published:** 2019-06-20

**Authors:** Nishad Matange

**Affiliations:** Department of Biology, Indian Institute of Science Education and Research (IISER), Pune, India

**Keywords:** glycosome, inorganic polyphosphates, nucleus, polyphosphatase, trypanosomes

## Abstract

An explosion of sequence information in the genomics era has thrown up thousands of protein sequences without functional assignment. Though our ability to predict function based on sequence alone is improving steadily, we still have a long way to go. Proteins with common evolutionary origins carry telling sequence signatures, which ought to reveal their biological roles. These sequence signatures have allowed us to classify proteins into families with similar structures, and possibly, functions. Yet, evolution is a perpetual tinkerer, and hence, sequence signatures alone have proved inadequate in understanding the physiological activities of proteins. One such enigmatic family of enzymes is the NUDIX (**nu**cleoside **di**phosphate linked to a moiety **X**) hydrolase family that has over 80000 members from all branches of the tree of life. Though MutT, the founding member of this family, was identified in 1954, we are only now beginning to understand the diversity of substrates and biological roles that these enzymes demonstrate. In a recent article by Cordeiro et al. in Bioscience Reports [Biosci. Rep. (2019)], two members of this protein family from the human pathogen *Trypanosoma brucei* were deorphanized as being polyphosphate hydrolases. The authors show that of the five NUDIX hydrolases coded by the *T. brucei* genomes, TbNH2 and TbNH4, show *in vitro* hydrolytic activity against inorganic polyphosphate. Through classical biochemistry and immunostaining microscopy, differences in their substrate specificities and sub-cellular localization were revealed. These new data provide a compelling direction to the study of Trypanosome stress biology as well as our understanding of the NUDIX enzyme family.

## Introduction

The advent of the genomics era has upturned the pipeline of discovery in Biology. Modern day biochemists are now faced with the perplexing situation of being able to clone out novel genes and purify the proteins they code for with ease, but without any knowledge of their biological functions. In such cases, clustering of proteins in families with conserved sequences and/or structural motifs provides a first approximation of the kinds of substrates that an uncharacterized enzyme may utilize. Yet, we are far from being able to accurately predict protein function based on sequence information alone. This has resulted in extensive databases of protein families, in which, a majority of members remain unannotated and uncharacterized. One such large group of proteins is the NUDIX (**nu**cleoside **di**phosphate linked to a moiety **X**) hydrolase family. The Pfam database [1] has over 80000 sequences that have been classified as NUDIX hydrolases based on conserved sequence features. These identifying features, namely the GX_5_EX_7_REUXEEXGU motif, where U is a bulky aliphatic residue (such as leucine, isoleucine, or valine) and X is any amino acid, is referred to as the NUDIX box [2,3]. Residues of the NUDIX box chelate divalent metal ions, such as Mg^2+^, and thus, facilitate catalysis [4,5].

NUDIX hydrolases are ubiquitous across the tree of life, and the NUDIX box is typically found in multiple proteins in most bacterial and eukaryotic proteomes. The human genome, for instance, codes for 22 different NUDIX box-containing proteins [6]. The first NUDIX hydrolase to be discovered, and indeed the flagship member of this protein family, was MutT from *Escherichia coli*. Initially identified in a genetic screen for hypermutation phenotypes, careful biochemical and structural analyses have revealed that MutT and its functional homologs in other organisms possess diphosphatase activity. Their presence allows cells to ‘clean-up’ oxidized nucleotides, such as 8-oxo-2′-deoxyguanosine 5′-triphosphate, which can be mutagenic [2]. Thus, the primary role of NUDIX hydrolases was assumed to be in maintaining nucleotide homeostasis. As computation prediction and biochemical characterization of members of the NUDIX hydrolase family continues, it has become apparent that the substrate repertoire of this class of enzymes can range from ADP-sugar conjugates and NADH to 5′-caps on RNA and diphosphoinositol polyphosphates [3]. A recent study on human NUDIX hydrolases elegantly captured the variety of substrates that are hydrolyzed by these enzymes [6]. In fact, the presence of a pyrophosphate bond appears to be the only common theme among the molecules that can serve as substrates for these enzymes. Some NUDIX hydrolases have also evolved catalysis-independent functions. For instance, the human DBC1 protein, which contains a NUDIX-box, is catalytically inactive, but functions as a nucleotide-binding domain that regulates the activity of the Sirt1 deacetylase [7]. The spectrum of catalytic and non-catalytic activities demonstrated by NUDIX hydrolases has broken the stereotype of these proteins being part of the cell’s clean-up machinery. We now know that NUDIX hydrolases may participate in a plethora of cellular pathways, including RNA processing, sterol metabolism and tumor suppression [3].

## NUDIX hydrolases and inorganic polyphosphate metabolism in *Trypanosoma*

The identification of Ddp1p, a NUDIX family hydrolase from yeast, as an endopolyphosphatase that is active against inorganic polyphosphates has provided a new set of substrates for these enzymes [8]. Polyphosphates are polymers of varying lengths in which inorganic phosphate moeities are linked by high energy phosphoester bonds. These polymers are thought to be ancient molecules that may have served as energy sources on early earth [9,10]. They are found in all cellular organisms and are often localized to granules or sub-cellular compartments. In addition to their use as energy reserves, polyphosphates are known to be responsible for functions as diverse as cellular motility [11] and sporulation [12] in bacteria, to triggering apoptosis in higher eukaryotes [13].

Of particular interest is the role played by inorganic polyphosphates in the virulence of pathogens. For instance, *Pseudomonas aeruginosa*, a pathogenic bacterium, is attenuated upon depletion of its polyphosphate reserves. This attenuation has been attributed to dampened quorum sensing and biofilm formation [14]. Among eukaryotic pathogens, a role for polyphosphates in virulence is coming to the fore for protozoan parasites such as *Trypanosoma brucei*. Trypanosomiasis, also called African sleeping sickness, is endemic to 36 countries in sub-Saharan Africa and during epidemics, it can pose an even greater risk of mortality than HIV/AIDS (source: Trypanosomiasis Fact Sheet, World Health Organization). *T. brucei* – like other protozoan parasites – uses an insect vector, in this case the Tse-Tse fly, to infect humans. Alternating between insect and human hosts, *T. brucei* necessarily requires rapidly adapting to diverse stressful environments and appropriately shifting its metabolism. In this regard, inorganic polyphosphates and the enzymes that are involved in its metabolism seem to play an important role. Deletion of TbVtc4, a polyphosphate kinase that synthesizes polyphosphate, as well as TbVsp1, which is involved in the degradation of a polyphosphate, attenuates the parasite in infection models. Perturbation of polyphosphate metabolism also results in lower stress tolerance, in particular lower osmo-tolerance, than wild-type [15,16].

In a recent article by Cordeiro et al. in Bioscience Reports [17] two additional mediators of polyphosphate metabolism in *T. brucei* have been identified. Cordeiro et al. [17] begin by asking if any of the five NUDIX family enzymes from *T. brucei* are active against polyphosphates. Using a standard assay for polyphosphate degradation, they show that TbNH2, a hitherto uncharacterized enzyme, demonstrates robust exopolyphophatase activity *in vitro*. TbNH4, previously shown to have RNA decapping activity [18], also has polyphosphatase activity, while none of the other NUDIX hydrolases from *T. brucei* are able to use polyphosphate as a substrate. The deorphanizing of TbNH2 and TbNH4 adds to our understanding of polyphosphate metabolism in Trypanosomes and also demonstrates polyphosphatase activity in two more NUDIX family members. Interestingly, neither of these enzymes has a great deal of sequence similarity to the Ddp1p yeast polyphosphatase outside the NUDIX box. Further, the TbNH2 and TbNH4 enzymes demonstrate significant differences in their catalytic preferences. TbNH2 distinguished strongly between short and long polymers of polyphosphate, while TbNH4 showed only a marginal difference in catalytic efficiency toward these two substrates. TbNH2 also demonstrated activity against ADP and ATP, while TbNH4 did not. It remains to be seen what structural or sequence features these biochemical differences can be attributed to. However, it is clear that these differences in catalytic activity are indicative of different physiological roles of these enzymes. Cordeiro et al. also go on to show differences in sub-cellular localization of TbNH2 and TbNH4 proteins. While TbNH2 localized specifically to glycosomes, TbNH4 localized to the nucleus and cytosol. The localization of these two enzymes reiterates that their roles in the cell are most likely different [17].

## Spatio-temporal regulation of polyphosphate metabolism?

What are the implications of the findings presented by Cordeiro et al. [17]? TbNH2 and TbNH4 add to our understanding of polyphosphate metabolism in *Trypanosoma*. These enzymes may also provide us with new tools to perturb polyphosphate levels in *T. brucei*. This is essential given the role that inorganic polyphosphates play in the biology of these organisms. Further, TbNH2 has close orthologs in other related kinetoplasts like *Leishmania*, where it may also regulate polyphosphate levels. More importantly, the presence of multiple, spatially separated polyphosphatases in *T. brucei* suggests that the levels of inorganic polyphosphates are exquisitely regulated in different compartments of the cell. Traditionally, polyphosphates were thought to be synthesized and retained in acidocalcisomes. Inorganic polyphosphates are now known to be present in the glycosome and nucleolus of *T. brucei* as well [19]. In other eukaryotes too, polyphosphates have been detected in the cytosol [20], nucleus [21] and mitochondria [22]. The fact that *T. brucei* differentially localizes polyphosphate-metabolizing enzymes to these different compartments indicates the importance of spatio-temporal regulation in the levels of this polymer.

Polyphosphates, in addition to serving as stores of inorganic phosphates, are emerging multi-functional molecules [9], and tissue/sub-cellular localization is an important regulatory mechanism employed by living systems to regulate their influence. For instance, extracellular Ca^2+^ ion chelation by polyphosphates inhibits bone mineralization in mammals [23]. Growth phase-dependent nuclear accumulation of polyphosphate has been reported in yeast cells [21]. Polyphosphates in the mitochondria of cardiomyocytes are known to modulate the opening of the mitochondrial permeability transition pore [24]. Similarly, mitochondrial polyphosphates in astrocytes actively modulate cellular metabolism [25]. In *T. brucei* as well as *T. cruzi*, it was recently demonstrated that glycosome-localized polyphosphates interact with many glycolytic enzymes, consistent with the view that inorganic polyphosphates can act as scaffolds for protein interactions [19,26]. The glycosome, a specialized peroxisome that harbors glycolytic enzymes, is an essential organelle for *Trypanosoma* as bloodstream forms of the parasite rely almost exclusively on the glycolysis for their energetic requirements [27]. Similarly, nucleolus-localized polyphosphates interact with ribosomal proteins as well as master-regulator kinases [19]. The functional consequences of these interactions remain to be determined, but it is clear that polyphosphatases like TbNH2 and TbNH4 may differentially influence glycosomal and nucleolar proteins; thus, modulating Trypanosomal biology differently. Finally, for TbNH4, which also has RNA decapping activity [18], polyphosphate hydrolysis may itself be a mechanism that regulates enzyme activity against other substrates. These and other possible roles for trypanosomal NUDIX polyphosphatases will no doubt open up new avenues for understanding the biology of *T. brucei* and possibly also provide new targets for therapeutic intervention in the future.

## Abbreviations

NUDIX: **nu**cleoside **di**phosphate linked to a moiety **X**
